# Determining the effective coverage of maternal and child health services in Kenya, using demographic and health survey data sets: tracking progress towards universal health coverage

**DOI:** 10.1111/tmi.12841

**Published:** 2017-02-07

**Authors:** Peter K. Nguhiu, Edwine W. Barasa, Jane Chuma

**Affiliations:** ^1^ Health Economics Research UnitKEMRI – Wellcome Trust Research ProgrammeNairobiKenya; ^2^ Nuffield Department of MedicineUniversity of OxfordOxfordUnited Kingdom; ^3^ The World BankKenya Country Office

**Keywords:** effective coverage, quality, maternal and child health, equity, Kenya, couverture effective, qualité, santé maternelle et infantile, équité, Kenya

## Abstract

**Objectives:**

Effective coverage (EC) is a measure of health systems’ performance that combines need, use and quality indicators. This study aimed to assess the extent to which the Kenyan health system provides effective and equitable maternal and child health services, as a means of tracking the country's progress towards universal health coverage.

**Methods and results:**

The Demographic Health Surveys (2003, 2008–2009 and 2014) and Service Provision Assessment surveys (2004, 2010) were the main sources of data. Indicators of need, use and quality for eight maternal and child health interventions were aggregated across interventions and economic quintiles to compute EC. EC has increased from 26.7% in 2003 to 50.9% in 2014, but remains low for the majority of interventions. There is a reduction in economic inequalities in EC with the highest to lowest wealth quintile ratio decreasing from 2.41 in 2003 to 1.65 in 2014, but maternal health services remain highly inequitable.

**Conclusions:**

Effective coverage of key maternal and child health services remains low, indicating that individuals are not receiving the maximum possible health gain from existing health services. There is an urgent need to focus on the quality and reach of maternal and child health services in Kenya to achieve the goals of universal health coverage.

## Abstract (fr)

**Objectifs:**

La couverture effective est une mesure de la performance des systèmes de santé, qui combine le besoin, l'utilisation et la qualité. Cette étude visait à évaluer dans quelle mesure le système de santé kenyan fournit des services de santé maternelle et infantile effectives et équitables, afin de pouvoir suivre les progrès du pays vers une couverture universelle de santé.

**Méthodes et résultats:**

Les enquêtes démographiques et de santé (2003, 2008‐2009 et 2014) et les enquêtes sur l’évaluation de la prestation des services (2004, 2010) ont été les principales sources de données. Les indicateurs de besoin, d'utilisation et de qualité de 8 interventions de santé maternelle et infantile ont été regroupés selon les interventions et les quintiles économiques pour calculer la couverture effective. La couverture effective est passée de 26,7% en 2003 à 50,9% en 2014, mais reste faible pour la majorité des interventions. Il y a eu une réduction des inégalités économiques dans la couverture effective avec le ratio entre le quintile de richesse le plus élevé et le plus bas, passant de 2,41 en 2003 à 1,65 en 2014, mais les services de santé maternelle demeurent très inéquitables.

**Conclusions:**

La couverture effective des services de santé maternelle et infantile clés reste faible, ce qui indique que les gens ne reçoivent pas le maximum possible des bienfaits de la santé grâce aux services de santé existants. Il est urgemment nécessaire d'améliorer la qualité des services de santé maternelle et infantile au Kenya pour atteindre les objectifs d'une couverture universelle de santé.

## Abstract (es)

**Objetivos:**

La cobertura efectiva es una medida del desempeño de los sistemas sanitarios que combina necesidad, uso y calidad. Este estudio tenía como objetivo evaluar el alcance del sistema sanitario de Kenia para proveer servicios de atención materno‐infantil efectivos y equitativos, como una forma de trazabilidad del progreso del país hacia una cobertura sanitaria universal.

**Métodos y Resultados:**

Los Censos Demográficos y Sanitarios (2003, 2008‐09 and 2014) y las Encuestas de Evaluación de Provisión de Servicios (2004, 2010) fueron las principales fuentes de datos. Los indicadores de necesidad, uso y calidad de 8 intervenciones sanitarias materno‐infantiles fueron agregadas a través de intervenciones y quintiles económicos para calcular la cobertura efectiva. La cobertura efectiva ha aumentado del 26.7% en el 2003 al 50.9% en el 2014, pero continúa siendo baja para la mayor parte de las intervenciones. Hay una reducción en las inequidades económicas en la cobertura efectiva, con la ratio del mayor a menor quintil de riqueza disminuyendo de 2.41 en el 2003 al 1.65 en el 2014, pero los servicios de salud materna continúan siendo altamente injustos.

**Conclusiones:**

La cobertura efectiva de los principales servicios de salud materna e infantil continúa siendo baja, indicando que los individuos no están recibiendo las máximas ganancias posibles en salud de los servicios sanitarios existentes. Existe una necesidad urgente de mejorar la calidad de los servicios de salud materno‐infantil en Kenia para conseguir los objetivos de la cobertura sanitaria universal.

## Introduction

Since the 58th WHO Assembly resolution 1 and the 2010 World Health Report 2, there has been increased focus on universal health coverage (UHC) – defined as a situation where the entire population has access to needed healthcare services, of good quality to be effective, without undue financial hardship 1, 3. The importance of UHC has recently been demonstrated by its inclusion as one of the Sustainable Development Goals (SDG). SDG 3.8 urges countries to ensure protection of their population from financial risk arising from seeking care, while also ensuring access to safe, effective, quality and affordable care 4.

To ensure that Kenya's citizens realise their constitutional rights to health 5, the government is implementing a wide range of health financing reforms. For the first time in Kenya's history, the government allocates resources every year to compensate health facilities for revenue losses arising from user fee removal. These conditional allocations amounted to KES 900 million (USD: 9 million) for free primary healthcare services and KES 4.3 billion (USD: 43 million) for free maternity services in the financial year 2015/2016 budget 6. Other initiatives include the civil servants’ health insurance scheme introduced in 2012, which provides comprehensive cover to all civil servants and their dependents, and a full health insurance subsidy for the poor, elderly and disabled population through the National Health Insurance Fund (NHIF). While these initiatives are important developments for Kenya, they largely focus on providing financial risk protection which is of no value unless good quality services are accessible when needed. The Ministry of Health has adopted the Kenya Quality Model for Health 7 as the national quality assurance framework. Although this framework incorporates indicators for tracking implementation through monitoring 12 domains, many of the indicators are structural and point to availability of resources or capacity to provide services. The large list containing more than 100 indicators has also proven difficult to administer resulting in slow uptake of KQMH, with only a few programmes and departments making headway in development and implementation of standards of care 8, provider accreditation 9 and enforcement of purchasing contracts 10.

The UHC monitoring framework developed by WHO and World Bank 11 proposes that measurement of coverage with financial risk protection be conducted simultaneously with measurement of coverage with essential prevention and treatment services, indicators of which often include a quality component referred to as effective coverage (EC). Ng and colleagues 12 have noted that EC assesses the performance of a health system by measuring the extent to which healthcare services deliver their potential health gains to the population. EC not only measures the proportion of the population that make contact with the service of interest (which has previously been referred to as ‘contact coverage’) 11, 13, but more importantly adjusts this proportion using a weight that reflects the quality of service being offered. It is calculated at individual level for each indicator service and aggregated to give the population‐level EC. The indicator services can be adapted to a country's needs and priorities, thereby allowing countries to focus their efforts on the services that are lagging behind either in reach or quality. As EC can be estimated for subpopulations of interest, it is also useful in estimating socio‐economic and geographic inequalities 14. This study aimed to estimate the levels of and inequities in EC of maternal and child health (MCH) services in Kenya, as a means of tracking the country's progress towards UHC.

## Methods

### Data sources

Data were obtained from two sets of nationally representative surveys: the Kenya Demographic and Health Surveys (KDHSs) 15, 16, 17 and the Kenya Service Provision Assessment (KSPA) Surveys 18, 19. The KDHS is a household‐based survey conducted every 5 years to collect data on marriage, fertility, family planning, reproductive health and child health 20. The KDHS incorporates a two‐stage sampling design, with the primary sampling units being clusters drawn via probability proportional to size sampling, from enumeration areas defined during the national census and spread across the country. At the second stage, 25 households are selected by equal probability sampling from a household list in each selected cluster. Further details on each survey's sampling strategy are obtained from the respective survey report.

Three rounds of KDHS (2003, 2008*–*2009 and 2014) were analysed. A total of 8195 women and 6102 children aged under 5 years were included in the 2003 KDHS analysis. In 2008*–*2009, there were 8444 women and 5852 children. In 2014, more clusters and households were sampled, and therefore, there were 31 079 women and 19 563 children under 5 years. The data set provided population‐level need and utilisation data for MCH services and some proxy measures of quality.

The KSPA is a formal health facility survey designed to provide information on the delivery of reproductive, maternal, newborn and child health services. Facilities are assessed for availability of structural features (equipment, infrastructure and medicines) and supportive processes (such as client recordkeeping, and adherence to guidelines and standards of care) 21. The KSPA randomly samples facilities from Kenya's Master Facility List which incorporates all formal and functioning health facilities at the time of the survey. Sampling is stratified by region and by type of facility (hospital, health centre, maternity, dispensary, clinic and stand‐alone voluntary counselling and testing facilities). Further details on each survey's sampling strategy are obtained from the respective survey report. There were a total of 440 (97.1% of sampled) facilities surveyed in the 2004 KSPA and 695 (98.8% of sampled) facilities surveyed in the 2010 KSPA, respectively. These data were analysed to estimate intervention quality and for the calculation of EC for four of the MCH services.

### Data analysis

Estimating the level and distribution of EC involves four key steps: identifying interventions for assessment; defining measures of need, use and quality; estimating individual and population‐level EC; and assessing inequalities in EC.

For the identification of key interventions, eight MCH services were identified for assessment as shown in Table 1. These interventions were selected from the recommendations of the Commission on Information and Accountability for Women and Children's Health Report 22 based on their relevance to national priorities and on availability of data from the KDHS survey data sets.

**Table 1:** Definition of indicators for estimating EC

MCH service	Measure of need (denominator)	Measure of use (numerator)	Quality estimator
Family planning services	Women 15–49 years old who at the time of survey were able to get pregnant (fecund)	Fecund women 15–49 years old, currently using a modern contraceptive method	Facility level score based on the presence of client privacy during consultation, availability of reproductive health counselling visual aids and record tools, and reproductive health commodity management practices in a facility. (source KSPA)
Functional antenatal services	Women 15–49 years old with at least one child under 5 years	Women 15–49 years old with at least one child under 5 years, whom for their most recent birth, reported having made at least four visits for ANC	Individual level score if the respondent recalls the following services being performed: blood pressure taken, urine sample taken, blood sample taken, respondent informed about pregnancy complications, iron tablets/syrup prescribed, and a drug for intestinal parasites prescribed, during any ANC visit (source KDHS)
Skilled delivery and perinatal care	Women 15–49 years old with at least one child under 5 years	Women 15–49 years old with at least one child under 5 years, whom for their most recent birth, reported attendance by a skilled health provider (doctor, nurse or midwife)	Facility level score based on reported routinely performed essential new‐born care practices at the facility maternity. These included routine rooming in with the mother, routine weighing of new‐borns, complete examination of new‐borns before discharge, administration of BCG before discharge and other indicators. (source KSPA)
Breastfeeding during the first 6 months of life	All children between 0 and 5 months	All children between 0 and 5 months, for whom breastfeeding was reported in the preceding 24 h	Individual level: All children between 0 and 5 months for whom exclusive breastfeeding (breastfeeding only, with no other complementary feed offered) was reported in the preceding 24 h
Immunisation services	All children alive between 12 and 23 months	All children alive between 12 and 23 months who received the complete set of vaccines as outlines in the Kenya Ministry of Health National Vaccination Schedule i.e. BCG, three doses of oral or intravenous Polio, three doses of Diphtheria, Pertussis, Tetanus, Hepatitis B and Hemophilus Influenza type B pentavalent vaccine, three doses of pneumococcal vaccine (from Jan 2011 onwards), and Measles vaccines	Facility level score based on observed or health worker reported availability of at least one working weighing scale and thermometer, and routinely performed processes including use of guidelines to assess and treat sick children, routine weighing, temperature taking and recording, assessment of immunization status and keeping of individual patient records. (source KSPA)
Management of diarrhoea	All children under 5 years reported to have had diarrhoea in the preceding 4 weeks	All children that had diarrhoea in the preceding 4 weeks, who were given oral rehydration therapy (ORT) or increased fluids.	Individual level: Proportion of children who had diarrhoea in the preceding 4 weeks, who were given the guideline recommended oral rehydration salt mixture
Care seeking for acute respiratory illness and/or fever	All children under 5 years reported to have had acute respiratory illness and/or fever in the preceding 2 weeks	All children who had acute respiratory illness and/or fever, for whom advice on treatment was sought from a medical provider	Facility‐level score based on observed or health worker reported availability of at least one working weighing scale and thermometer, and routinely performed processes including use of guidelines to assess and treat sick children, routine weighing, temperature taking and recording, assessment of immunisation status and keeping of individual patient records (source KSPA)
Use of insecticide ‐treated nets	All children and pregnant women	Proportion of children and pregnant women living in household that owned an ITN	Individual level: Proportion of children and pregnant women who actually slept under an insecticide‐ treated net in the preceding night

EC, effective coverage; MCH, maternal and child health.

While contact coverage is estimated as a ratio of the target population receiving an intervention, EC is estimated as 23

$${\text{EC}}_{ij}=Q_{ij}\times U_{ij}\;|\;N_{ij}=1$$

where EC_*ij*_ is effective coverage for an individual *i* receiving intervention *j*,* Q* is the proportion of potential health gain that is achieved from the intervention, and *U* is the probability of receiving the intervention conditional on need (the contact coverage of the intervention). A dichotomous classification of *N* was used where need is identified as either existent (*N* = 1) or non‐existent (*N* = 0) for each individual in the data set, with regard to each of the eight interventions assessed.

Estimating quality of care using household surveys is always difficult and data are prone to several limitations. In this study, *Q* was estimated from components of the content of care as has been described by Ng *et al*. 12, for four interventions (antenatal services, breastfeeding during the first 6 months of life, management of diarrhoea and use of insecticide‐treated nets). This was based on reports of respective counselling received, clinical and screening tests performed, medication or commodities received, as recommended by the respective service guidelines (Table 1). For instance, the quality of antenatal services was estimated by assigning a score to each of the following if reportedly performed on the respondent: blood pressure taken, urine sample taken, blood sample taken, respondent informed about pregnancy complications, iron tablets/syrup prescribed and a drug for intestinal parasites prescribed.

There were four interventions (family planning, skilled delivery and perinatal care, immunisation and care seeking for acute respiratory illness or fever) for which *Q* was estimated not at individual level, but at a regional (provincial) level. Estimates were obtained from the KSPA data sets, and an average provincial score for each intervention was computed. The KDHS and KSPA data sets were then linked at the provincial level based on the survey domain method described by Burgert and Prosnitz 24 as they shared the same boundaries. Further information on the actual construction of respective quality indicators is detailed in Appendix S1.

To estimate population‐level effective coverage (*EC*_*j*_) for each specific intervention *j*, the (survey weighted) average of individual EC was calculated. These intervention‐specific EC values were then averaged to give an overall EC estimate. Taylor series approximation 25 was used to calculate the standard errors (Appendix S1).

To assess economic inequalities in EC, households were grouped into quintiles and their EC was computed using the asset‐based wealth index in the DHS 26. Comparisons were made between individuals in the highest and lowest quintiles, by calculating the high‐to‐low ratio of EC. This measure, although simple and intuitive to understand, assesses differences between the two ends of the economic hierarchy and does not make use of the rest of the population's information. Therefore, the relative concentration index 27 was also computed to correlate EC with the wealth asset ranking of individuals.

Data analysis was performed in r version 3.3.2 (The R Foundation for Statistical Computing, Vienna, Austria) and involved population‐weighted aggregation of EC across individuals and interventions.

## Results

The demographic characteristics across the three survey periods are detailed in Table 2. There were no major differences in characteristics noted, with the exception that in 2014, women with secondary or higher education were proportionally more than in 2008*–*2009 or 2003. The KDHS utilises a sampling design that stratifies samples along regional (provincial) and urban/rural strata. While previous surveys have worked with a rural–urban ratio of 3:1, in 2014, the survey sampled rural and urban households at a ratio of 3:2 resulting in a higher proportion of urban households in 2014.

**Table 2:** Description of KDHS survey respondents’ characteristics

	Group	2014	2008*–*09	2003
Women 15–49 years	*n*	31 079	8444	8195
Age group	15*–*19	18.7%	20.9%	22.6%
	20*–*24	18.5%	20.3%	20.6%
	25*–*29	19.6%	17.2%	16.9%
	30*–*34	14.5%	14.3%	13.3%
	35*–*39	12.1%	10.4%	10.6%
	40*–*44	9.3%	9.1%	9.6%
	45*–*49	7.3%	7.8%	6.4%
Highest education level	No education	7.0%	8.9%	12.7%
	Primary	50.3%	56.8%	58.0%
	Secondary	31.5%	26.9%	23.5%
	Higher	11.2%	7.4%	5.9%
Children <5 years	*n*	19 563	5852	6102
Gender	Male	50.8%	51.7%	51.0%
	Female	49.2%	48.3%	49.0%
Age of child	<1 year	18.4%	19.5%	20.5%
	1–<2 years	19.3%	18.7%	18.5%
	2–<3 years	19.2%	19.3%	16.9%
	3–<4 years	19.9%	18.3%	18.4%
	4–<5 years	18.8%	17.8%	16.8%
Households	*n*	24 565	6430	6159
Asset‐based wealth quintile	Poorest	16.3%	16.9%	17.7%
	Poorer	17.7%	17.2%	18.4%
	Middle	19.1%	18.8%	18.5%
	Richer	21.5%	20.5%	20.1%
	Richest	25.5%	26.7%	25.3%
Geographic region	Urban	41.1%	25.7%	24.6%
	Rural	58.9%	74.3%	75.4%

### Effective coverage levels and trends

There has been improvement from 2003 to 2014 in almost all indicators. Although the aggregate EC level of MCH increased from 26.7% in 2003 to 50.9% in 2014, it remains quite low and approximately half of the population still do not receive MCH services to the required standards of care. In 2014, EC was higher for some interventions: management of diarrhoea – 53.8% (CI: 51.2–56.4%), breastfeeding during the first 6 months of life – 71.6% (CI: 67.7–75.5%), and use of insecticide‐treated nets – 59.0% (CI: 57.4–60.6%). Facility‐level health services reflected lower ECs across board: family planning – 40.7% (CI: 39.7–41.7%), antenatal services – 44.6% (CI: 43.2–46.0%), skilled delivery and perinatal care – 51.3% (CI: 49.8–52.8%), and care seeking for acute respiratory illness or fever – 41.1% (CI: 39.5–42.7%). Table 3 shows the levels of contact and EC for all tracer interventions. The gap (median 21.0, range 10.5–28.0 in percentage points) between contact and EC reflects missed opportunities for delivering health gains to the population due to the quality of the interventions being offered.

**Table 3:** Table of contact coverage and EC of maternal and child health: 2014, 2008*–*2009 and 2003

MCH service	Indicator		2014		2008*–*09		2003	
			% (95% CI)	Number in need (*n*)	% (95% CI)	Number in need (*n*)	% (95% CI)	Number in need (*n*)
Family planning services	Currently using a modern family planning method	Contact coverage	67.8 (66.4–69.2)	10 535	62.0 (60.0–64.0)	5760	53.7 (51.8–55.6)	5159
		EC	40.7 (39.7–41.7)		36.6 (35.3–37.9)		31.9 (30.3–33.5)	
Functional antenatal services	Attended four or more ANC visits during last pregnancy	Contact coverage	58.2 (56.5–59.9)	6865	47.1 (44.7–49.5)	3973	52.3 (50.2–54.4)	4051
		EC	44.6 (43.2–46.0)		31.6 (29.8–33.4)		31.7 (30.0–33.4)	
Skilled delivery and perinatal care	Most recent birth attended to by skilled health provider	Contact coverage	61.8 (60.3–63.3)	19 563	43.8 (40.5–47.1)	5851	41.6 (39.1–44.1)	6102
		EC	51.3 (49.8–52.8)		36.7 (35.0–38.4)		32.6 (30.8–34.4)	
Breastfeeding during the first 6 months of life	Exclusive breastfeeding	Contact coverage	99.6 (99.2–100)	800	99.1 (98.4–99.8)	534	99.8 (99.6–100)	620
		EC	71.6 (67.7–75.5)		46.1 (41.0–51.2)		68.0 (63.6–72.4)	
Immunisation services	Received complete set of basic vaccines	Contact coverage	80.1 (78.5–81.7)	3865	76.5 (72.9–80.1)	1150	59.3 (55.8–62.8)	1193
		EC	55.6 (53.8–57.4)		54.1 (51.6–56.6)		34.4 (32.1–36.7)	
Management of diarrhoea	Given ORT for management of most recent bout of diarrhoea	Contact coverage	81.8 (80.0–83.6)	2843	77.2 (73.1–81.3)	909	76.7 (73.2–80.2)	888
		EC	53.8 (51.2–56.4)		38.8 (33.9–43.7)		29.2 (25.4–33.0)	
Care seeking for acute respiratory illness and/or fever	Sought medical advice for most recent episode of fever/ARI	Contact coverage	58.5 (56.6–60.4)	6102	47.7 (43.8–51.6)	1490	45.5 (42.4–48.6)	2496
		EC	41.1 (39.5–42.7)		33.0 (30.3–35.7)		25.5 (23.7–27.3)	
Use of insecticide‐treated nets	Covered with insecticide‐treated net	Contact coverage	75.3 (73.9–76.7)	19 664	72.6 (68.5–76.7)	5756	22 (19.8–24.2)	5870
		EC	59.0 (57.4–60.6)		53.8 (49.9–57.7)		7.0 (5.9–8.1)	
Aggregated		Contact Coverage	68.1	77 816	59.0	25 427	45.0	26 381
		EC	50.9		40.6		26.7	

ANC, Antenatal care; ARI, Acute respiratory illness; EC, Effective coverage; MCH, maternal and child health; ORT, Oral rehydration therapy.

### Distribution of EC across economic groups

**Figure 1:**
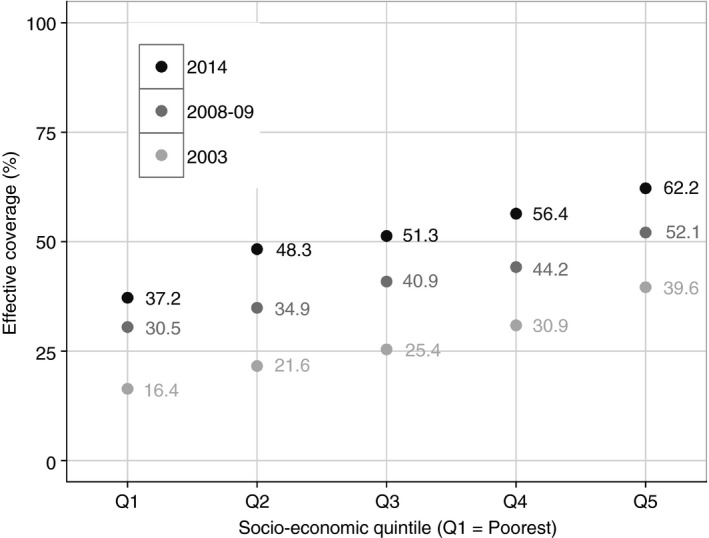
Effective coverage of maternal and child health services in Kenya.

With the increase in aggregate EC (Figure 1) over the analysis period, there has been a general reduction in the economic inequalities in EC for MCH services. Table 4 shows the economic inequalities in EC from 2003 through 2014, using the high‐to‐low ratio (i.e. the ratio of EC between the wealthiest and the poorest quintiles) and the relative concentration index for each intervention (in this manuscript, we propose to abbreviate this as CIX in order to avoid any confusion with the abbreviation of the confidence interval). The high‐to‐low ratios of the indicator services are all declining towards 1 – with the aggregate EC high‐to‐low ratio having reduced from 2.41 in 2003 to 1.67 in 2014. On the contrary, management of diarrhoea has consistently been pro‐poor with a CIX of −0.088 in 2003 and −0.173 in 2014, showing that high contact coverage does not necessarily promote inequities if implemented in a pro‐poor manner.

**Table 4:** Socio‐economic distribution of EC, and relative Concentration Indices for MCH interventions in 2014, 2008*–*2009 and 2003

MCH Service		2014		2008*–*09		2003	
		High‐to‐low ratio	Concentration index	High‐to‐low ratio	Concentration index	High‐to‐low ratio	Concentration index
Family planning services	Currently using a modern family planning method	1.54	0.059 (0.046 to 0.072)	1.69	0.067 (0.047 to 0.087)	1.82	0.084 (0.061 to 0.106)
Functional antenatal services	Attended four or more ANC visits during last pregnancy	1.92	0.051 (0.025 to 0.077)	2.31	0.100 (0.066 to 0.133)	2.38	0.086 (0.055 to 0.118)
Skilled delivery and perinatal care	Most recent birth attended to by skilled health provider	3.05	0.209 (0.199 to 0.219)	4.05	0.267 (0.239 to 0.295)	4.76	0.280 (0.255 to 0.305)
Breastfeeding during the first 6 months of life	Exclusive breastfeeding	1.18	−0.095 (−0.124 to −0.065)	0.95	−0.120 (−0.182 to −0.058)	0.88	−0.096 (−0.437 to 0.245)
Immunisation services	Received complete set of basic vaccines	1.40	0.067 (0.053 to 0.080)	1.30	0.051 (0.013 to 0.090)	1.61	0.097 (0.060 to 0.134)
Management of diarrhoea	Given ORT for management of most recent bout of diarrhoea	1.05	−0.173 (−0.200 to −0.146)	0.92	−0.157 (−0.230 to −0.084)	1.20	−0.088 (−0.161 to −0.015)
Care seeking for acute respiratory illness and/or fever	Sought medical advice for most recent episode of fever/ARI	1.19	0.024 (0.003 to 0.044)	0.96	−0.006 (−0.053 to 0.041)	1.31	0.062 (0.024 to 0.101)
Use of insecticide treated nets	Covered with insecticide−treated net	1.38	−0.026 (−0.048 to −0.014)	1.35	−0.031 (−0.094 to −0.013)	7.52	0.281 (0.188 to 0.374)
Aggregate distribution across interventions		1.67		1.71		2.41	

ANC, Antenatal care; ARI, Acute respiratory illness; EC, Effective coverage; MCH, maternal and child health; ORT, Oral rehydration therapy.

Of the four interventions with the highest inequality in distribution of EC, skilled delivery and perinatal care, immunisation services, antenatal care and reproductive health/family planning, three are specifically maternal health services. In 2014, the wealthiest quintile had about twice the EC of ANC services than the poorest quintile (CIX: 0.051). Inequalities were highest for skilled delivery/perinatal care, where the wealthiest quintile had three times greater EC than the poorest quintile (CIX: 0.209). Use of ITNs was highly inequitable in 2003 (CIX: 0.281) but less so in 2014 (CIX: −0.026).

## Discussion

### Overall EC of MCH services has steadily increased over time

We set out to calculate EC levels for selected MCH services in Kenya, by multiplying contact coverage with a quality score computed from the content of care provided. We found that there has been improvement in all indicators. Both the overall EC (50.9% in 2014, 40.6% in 2008*–*2009 and 26.7% in 2003) and the overall contact coverage (45.0–68.1%) of MCH services have steadily increased over the period of analysis. The increase in coverage is supported by the Kenya Household Health Expenditure and Utilization Survey 2013 report which shows high levels of care seeking (77.8%) among household members who perceive themselves to be unwell 28. This increase could be attributed in part to some changes that have happened in the same period. First of all, there has been an overall increase in per capita total health expenditure from KES 3504.6 in 2001/2002 to KES 5679.5 in 2012/2013 29. Second, the construction of lower level health facilities over the period 2003–2007 resulted in an increase in the proportion of the population living within 5 kilometres of a public health facility from 71% in 2003 to 89% in 2008 30. At the same time, the percentage of the population living in urban areas in Kenya has increased from 19% in 1999 to 31.3% in 2009 31, 32, and urban areas are more likely to have health facilities manned by skilled health personnel who can deliver a wider range of maternal and child health services.

On the other hand, the constant gap between contact coverage and EC indicates that there is a need to focus on the quality of MCH services being delivered as Kruk *et al*. 33 have urged in a recent global health commentary, even as commensurate efforts are placed to increase service reach across underserved subpopulations.

Attempts to compare the EC computed with findings from other few studies are complicated by the different measures of quality that have been used. An assessment of EC of ANC across 41 countries, using DHS data from 2005 to 2011, found low coverage with specific antenatal content of care in all but eight countries 34. The authors obtained an average coverage of 54% from the 2008*–*2009 KDHS data set, by averaging the population coverage of individual processes of ANC care. Martinez *et al*. in 2011 35 found that in Central and South America, EC of deliveries by skilled birth attendants ranged from 56.4% in Haiti to 98.3% in the Dominican Republic. Here, the quality component was measured in terms of proportions of complications reported during delivery, and proportion of low birthweight deliveries occurring, but as these incidences are rare, the measure of quality may have been rather optimistic. In Mexico, in 2005*–*2006, the EC of deliveries with skilled birth attendants was 93.3% based on a binary quality measure indicating whether the delivery was conducted in a hospital, which was again more optimistic and cannot be directly compared with our estimate. Other different EC estimates have been computed in other low‐ and middle‐income countries related to different interventions: in trauma and obstetric surgical care 36, in voluntary counselling and testing services at major health facilities 37 and in treatment of hypertension 38.

### Inequality in coverage is reducing, but is still persistent for some maternal health services

From the results, inequality in overall EC of MCH services reduced between 2003 and 2014. Previous work has also demonstrated reduction in inequalities as coverage of specific MCH services such as vaccination and ITN use increased in Kenya, attributed largely to the government's scale‐up strategies through mass vaccination campaigns 39 and provision of free ITNs to children in all malaria‐endemic regions 40, 41. This is commendable considering that one of the dangers of poorly monitored efforts to increase coverage is a disproportionate benefit to the wealthier quintiles at the expense of the poorer quintiles 42, 43.

Despite this achievement, certain interventions still present inequality in distribution, such as skilled attendance, antenatal care and family planning. Consistent with this, Barros *et al*. in 2012 44 has highlighted varying inequalities in contact coverage with MCH services. In that study, delivery with a skilled birth attendant and contact coverage with four or more ANC visits demonstrated the largest inequality with concentration indices of 0.243 (95% CI: 0.138–0.296) and 0.172 (95% CI: 0.075–0.270), respectively, while contact coverage with early start of breastfeeding had the lowest inequality with a concentration index of 0.015 (95% CI: −0.017–0.047). In comparison, management of diarrhoea has remained distributed in a manner favouring the poor, with a CIX of −0.088 in 2003 and −0.173 in 2014, showing that high contact coverage does not necessarily promote inequities if implemented in a pro‐poor manner.

Large‐scale implementation of complementary community‐based interventions such as the Birth Preparedness Program (BPP) improved acceptability and reduced inequity in coverage with hospital delivery in Nepal 45. The implications of recent health financing reforms such as the increased per capita health expenditure, the directive on removal of maternity fees 46 and on the patterns of inequality in ANC and delivery with a skilled birth attendant require further assessment to examine their contribution.

## Limitations

One of the limitations of estimating EC level for MCH services in Kenya is that there are few other reliable data sources apart from the DHS. For instance, the multiple indicator cluster surveys (MICS) provide specific child health and nutrition interventions, but the recent surveys were conducted in selected regions only and were not country representative. On a positive note, the government of Kenya recently conducted an extensive survey on non‐communicable diseases and a TB prevalence study. This may allow for the expansion of estimates of EC beyond the traditional MCH indicators in line with future health policy direction. Further, the increase in use of electronic medical records could improve accuracy of routinely reported data by reducing data entry and computation errors during submission of monthly reports. This would provide a more efficient source of quality measures such as vaccination timeliness, obstetric and neonatal medicine consumption or even laboratory and radiology test results from ANC clinics, to improve on EC estimates. To closely monitor UHC, concerted efforts need to be placed across all sectors to increase demand, and to improve the use and quality of data at the point of care.

We acknowledge that there are challenges in estimating quality solely based on the components of care selected above. First, to estimate quality requires going beyond assessment of capacity to provide services (such as availability of job aids, weighing scales and thermometers). Taking the example of estimating EC of skilled delivery and perinatal care, Marchant and Schellenberg have shown that to obtain good estimates of the quality of care, health facility, health worker and household data have to be jointly interrogated 47. The Service Provision Assessment surveys are designed to capture data on both service readiness and actual processes of service execution, that is the approach we have used in our estimates of EC, for the interventions where the data were available.

Second, it may not always be possible to translate some of these measures to health gains (for instance, high scores in routine weighing of newborns, complete examination of newborns before discharge and administration of BCG before discharge may not easily be translated to lives saved at birth). However, these quality measures can be targeted for improvement based on their intrinsic value as levers for management action, making the metric useful for decision‐making at the policy level. To improve the resolution of measurement of quality, other designs such as mystery clients and patient follow‐up studies could supplement the data available especially for estimating the quality of family planning services, and the quality of medical care for children with acute respiratory illness or fever. Third, for the services whose quality measure was estimated from the KSPA, the quality measure was applied equally to all the individuals. The implication of this was a reduction in variance due to grouping of data at province level. This increases the confidence interval of the CIX estimates, meaning that it would be more difficult to detect true changes across the survey periods. Future work could consider geospatial methods to provide more resolute linkage, using the readiness and process execution indicators from a larger facility survey such as the service availability and reliability mapping survey, or even the routinely reported data from joint inspection of health facilities, if the data sets are made available.

Various approaches exist for aggregation of EC into an overall composite EC measure as described by Lozano *et al*. 48. Each intervention's EC could be aggregated with different weights either based on the overall possible health gain obtainable from the intervention 23, or based on preferences derived from stated choices from policymakers. We applied weights corresponding to the individual interventions’ denominator size in order to give interventions that were needed by a larger population more contribution in the overall EC estimate, although the overall results in this case did not differ significantly from computation of a simple average, due to the large denominators. Further work to establish policymakers’ preferences with regard to the computation of overall EC should be conducted to inform future EC estimation in Kenya. Nonetheless, the findings presented in this study provide a starting point for monitoring EC in Kenya. The study complements existing literature on measurment of EC for tracking UHC 12, 49, and monitoring of inequality in coverage with health services 44, 50.

## Conclusions

Although the levels of EC are improving and inequality has diminished between 2003 and 2014, the level of EC for MCH interventions remains low in Kenya. There is a need to focus on both the quality and the reach of MCH services to achieve the goals of UHC. The methods being employed currently for estimating EC are numerous, diverse and deserving of more attention from the international community to effectively monitor UHC.

## Supporting information


**Appendix S1**. Estimating the quality of interventions, for calculation of effective coverage.

**Appendix S2**. Data on specific components used to calculate EC is presented below for each survey period assessed.

**Figure S1a.** EC: Family planning services.

**Figure S1b.** EC: Functional antenatal services.

**Figure S1c.** EC: Breastfeeding during first 6 months.

**Figure S1d.** EC: Immunisation services.

**Figure S1e.** EC: Management of diarrhoea.

**Figure S1f.** EC: Care seeking for ARI/fever.

**Figure S1g.** EC: Use of ITNs.

**Figure S1h.** EC: Skilled delivery & perinatal care.
