# Supplementary material for: Determining the effective coverage of maternal and child health services in Kenya, using demographic and health survey data sets: tracking progress towards universal health coverage
Source: Trop Med Int Health. 2017 Feb 7;22(4):442–53. doi: 10.1111/tmi.12841 (PMC5396138; doi:10.1111/tmi.12841)
Supplement: Supplementary file 1 — Appendix S1. Estimating the quality of interventions, for calculation of effective coverage. [file TMI-22-442-s001.docx]

## Supplement 1

## A. Estimating the quality of interventions, for calculation of effective coverage

Effective coverage ($EC$) is given as the probability that individuals will receive some health gain from an intervention, given that they need it. This probability is weighted by the expected quality of the intervention. The formal definition of effective coverage is given as

$${EC}_{ij}=f\left( Q_{ij},U_{ij},N_{ij} \right)=Q_{ij}*U_{ij} | N_{ij}=1$$

for an individual $i$ receiving intervention $j$, where $Q$ is the expected quality of the intervention, and $U$ is the probability of receiving the intervention (which is the expected value of coverage of the intervention in the population in need $N$).

$Q_{ij}$ is determined for each individual, based in part on the individual’s choice of provider, and in part on the provider’s capacity to provide optimal intervention. It is a function of the care that can be provided by an individual provider k, and the probability that the individual would choose to go to this provider.

$Q_{ij}=f({HG}_{ijk}, P\left( U_{ijk} \right))$

But how do we measure the expected quality $Q$? In this paper we assume that the quality of an intervention exists on a continuous scale which we cannot observe. We can however observe some components of service as recommended by the respective clinical guidelines, from the content of care being provided at the time of the survey (1). $Q$ is then determined by the observer, taking a value based on the proportion of components of service correctly observed during the survey. For instance, in estimating the quality fraction of family planning / reproductive health below, the proportion of ten components of service defined below was calculated to estimate a value of $Q$, on the ratio scale between zero and one.

**a)** **Family planning /reproductive health quality fraction.** According to the Kenya National Family Planning Guidelines for Service Providers (2010) (2), in order for facilities to provide appropriate family planning (FP) and reproductive health services, clients should receive adequate counselling and education to dispel myths and misconceptions of specific methods and promote their choices, integrate counselling for FP with STI prevention including HIV testing, and contraceptives should be made available and stored in a manner to maintain their efficacy. The components of service in this assessment included ten features such as the presence of client privacy during consultation, availability of reproductive health counselling visual aids and record tools, and adequate reproductive health commodity management processes in a facility. These were obtained from the respective Kenya Service Provision Assessment (KSPA) survey variables, which are standardised across all surveys and are as listed in Table 1 (The SPA variables are recoded from the questionnaire and may not bear the same variable number as the questionnaires therefore the table includes a column indicating the specific question in the 2010 SPA questionnaire, and how the data was obtained). From these, the (survey weighted) expected value of Q was calculated based on the power to detect estimates at the first sub-national administrative (province) level and applied to all households within that level.

Table 1: Components of service considered in calculation of quality fraction for FP / RH services, obtained from standard variables in KSPA

| variable | Structural Features | SPA Questionnaire number and measurement approach |
| --- | --- | --- |
| V325 | privacy for fp consult | 310 (Observation) |
| v325a-e | visual aids (5 standard ones, for reproductive health counselling) | 311.01 – 05 (health worker report) |
|  | Process Related features |  |
| lv320a | contraceptives stored in fp area protected from sunlight | 354.03 (Observation) |
| lv320b | contraceptives stored in fp area off floor/protected from water | 354.02 (Observation) |
| lv320c | storage room in area free of rodent/pests | 354.04 (Observation) |
| V324,v312 | individual records for fp clients, **or** register for fp consult present | 314 (health worker report) |

**b)** **Antenatal services quality.** The quality of antenatal care (ANC) can be estimated by measuring the content of care received by an eligible individual in the course of pregnancy. The partnership for maternal and new-born health (3) specifies that good quality ANC should comprise early recognition and management of pregnancy related complications, health education and prevention, screening and control of underlying illnesses including STIs, and support for birth preparedness and healthy behaviour during pregnancy. The quality score for ANC sought to measure this using a total number of 6 routine care processes obtained from the respective Kenya Demographic and Health Surveys (KDHS) variables as are listed in Table *2*. From these, the expected value of Q (a survey-weighted proportion) was then calculated.

Table 2: Components of service considered in calculation of quality fraction for antenatal services

| variable | Routine Practices (ANC) |
| --- | --- |
| m42c | during pregnancy - blood pressure taken |
| m42d | during pregnancy - urine sample taken |
| m42e | during pregnancy - blood sample taken |
| m43 | told about pregnancy complications |
| m45 | during pregnancy, given or bought iron tablets/syrup |
| m60 | drugs for intestinal parasites given during pregnancy |

**c) Skilled** **delivery and perinatal care quality.** For effective delivery, the skilled attendant requires an enabling environment at the facility. There is need for appropriate infrastructure, and a continuum of care that is supported by adequate supplies, equipment, drugs, good management and supportive supervision. As an indicator of the facility’s capacity to support delivery and to offer perinatal care, 6 reported essential practices of new-born care were assessed and their presence scored. These were obtained from the KSPA datasets (plus an additional 3 that were available only in 2010 due to guideline changes) as listed in *Table 3* (The SPA variables are recoded from the questionnaire and may not bear the same variable number as the questionnaires therefore the table includes a column indicating the specific question in the 2010 SPA questionnaire, and how the data was obtained). From these, the expected value of Q (a survey-weighted proportion) was calculated at the first sub-national administrative (province) level, and applied to all households within that level.

Table 3: Components of service considered in calculation of quality fraction for skilled delivery and perinatal care

| variable | Structural Features | S PA Questionnaire number and measurement approach |
| --- | --- | --- |
| v506a | rooming in mother/newborn | 538.02 (health worker report) |
| v512 | register for delivery clients present | 512 (Observation + health worker report) |
|  | Process measures of care |  |
| v507b | newborn routinely weighed at birth | 539.07 (health worker report) |
| v506c | kangaroo mother care practice reeported *(SPA 2010 only)* | 539.01 (health worker report) |
| v507i | newborn dried and wrapped to keep warm *(SPA 2010 only)* | 539.02 (health worker report) |
| v507k | complete exam of newborn performed before discharge *(SPA 2010 only)* | 539.04 (health worker report) |
|  | Evidence of administration of key medicines |  |
| v507e | newborn given oral polio vaccine prior to discharge | 539.12 (health worker report) |
| v507f | newborn given BCG prior to discharge | 539.13 (health worker report) |
| v506b | vitamin A given to mother | 538.03 (health worker report) |

**d)** **Breastfeeding during the first 6 months of life**. Early supplementation with complementary foods is discouraged because it can expose infants to pathogens, and it competes with the infant’s suckling thereby reducing breast milk intake and production. Although one can argue whether this intervention actually reflects an individual choice, we hypothesise that the decision to exclusively breastfeed is the more difficult option for a mother, compared with supplemental feeding, that this decision is driven by availability and effective consumption of information on best feeding practices and that this information is delivered through the health system. Indeed, the Kenyan Ministry of Health through the National Policy on Maternal, Infant and Young Child Nutrition 2012 (4), now recommends strongly that children be exclusively breastfed during the first 6 months of life.

The definition of exclusive or predominant breastfeeding practice is all children between 0 and 5 months in the respective KDHS survey, for whom breastfeeding occurred in the last 24hrs and who consumed **none** of the liquid or solid supplements listed in Table *4* below:

Table 4: Features considered in calculation of quality fraction for infant feeding practices

| 2003 | ..in the last 24 hours, number of times given is 0 |
| --- | --- |
| m37f | times gave child commercially produced baby formula |
| m37h | times given tinned, powdered or fresh animal milk |
| m37l | times gave child other liquid |
| m37m | times given pumpkin, carrots, red/yellow yams, red sweet pot. |
| m37n | times given any green leafy vegetables |
| m37o | times given mango, papaya or other vitamin a rich fruits |
| m37p | times given other solid, semi-solid foods |
| m37q | times given food made from local grain |
| m37r | times given food made from local roots/tuber |
| m37u | times gave child other fruits/vegetables |
| m37v | times gave child meat, poultry, fish, shellfish, eggs |
| m37w | times gave child legumes (lentils, beans, peanuts) |
| m37x | times gave child cheese/yogurt |
| 2008_09 | ..in the last 24 hours, infant was not fed on any of the following: |
| v410a | gave child tea or coffee |
| v411 | gave child tinned/powder or fresh milk |
| v411a | gave child baby formula |
| v412a | gave child baby cereal |
| v412b | gave child other porridge/gruel |
| v414e | gave child bread, noodles, other made from grains |
| v414f | gave child potatoes, cassava, or other tubers |
| v414i | gave child pumpkin, carrots, squash (yellow or orange inside |
| v414j | gave child any dark green leafy vegetables |
| v414k | gave child mangoes, papayas, other vitamin a fruits |
| v414l | gave child any other fruits |
| v414o | gave child food made from beans, peas, lentils, nuts |
| v414p | gave child cheese, yogurt , other milk products |
| v414s | gave child other solid-semisolid food |
| v414t | gave child organ meats, fish, eggs, meats |

**e**) **Optimum quality for management of diarrhoea**. The main recommendation to prevent progression in severity of diarrhoea among children under 5 is prompt intake of appropriate fluids, and in Kenya the recommended fluid is oral rehydration salt (ORS). The optimal practice at community level should not only be to give ORS, but also to continue feeding and increase fluid intake. Investigating this would indicate the quality of health education provided to mothers on management of diarrhoea, and the extent to which this translates to practices. We therefore calculated the number of children with recently reported diarrhoea episodes in the KDHS datasets, that were given pre-packaged ORS (variable h13 / h13b).

**f**) **Quality of primary care for children**. We considered only facilities in the KSPA that offer sick child consults and routine vaccinations. These were assessed for the following 7 components of service: use of guidelines to assess and treat sick children, presence of at least one working weighing scale (child and/or infant) and thermometer, routine weighing, temperature taking and recording, assessing of immunization status and keeping of individual patient records for sick child consults as displayed in Table *5* (The SPA variables are recoded from the questionnaire and may not bear the same variable number as the questionnaires therefore the table includes a column indicating the specific question in the 2010 SPA questionnaire, and how the data was obtained). From these, the expected value of Q (a survey-weighted proportion) was calculated at the first sub-national administrative (province) level, and applied to all households within that level.

Table 5: Components of service for optimal primary care for children

| variable | Structural Criteria | SPA Questionnaire number and measurement approach |
| --- | --- | --- |
| v265b:c | infant weighing scale **or** child scale present | 257.01 – 02 (Observation) |
| v265d | Thermometer present | 257.05 (Observation) |
|  | Process Related Criteria |  |
| v247a | Child weigh taken | 251.01 (Observation + health worker report) |
| v247c | child's temperature taken | 251.03 (Observation + health worker report) |
| v247d | Assessment of immunization status | 251.04 (Observation + health worker report) |
| v263 | individual records/charts for sick child consults | 263 (Observation+ health worker report) |
| V242 | IMCI guidelines used to assess/treat sick children *(applies to 2010 SPA)* | 246 (health worker report) |

**g**) **Coverage with insecticide treated nets**. The revised Kenya Malaria Strategy (5) aims to attain universal coverage of ITNs, defined as reaching a distribution ratio of one ITN for every two people, in conjunction with increasing use of those nets to 80% in all areas with a malaria risk in the country by 2018. In order to assess the effectiveness of use of ITNs the proportion of individuals living in households that reportedly owned mosquito nets were asked whether they had slept under an ITN, an untreated net, or no net during the preceding night. Only those that reported to have slept under an ITN (variable ml101/ ml0) were considered to be effectively covered.

## B. Calculating the precision around effective coverage estimates: Taylor series approximation

In this analysis, the contact coverage is computed as a proportion of use, from the same household level survey (the Kenya Demographic and Health Survey). EC for four of the interventions studied (functional antenatal services, breastfeeding during the first 6 months of life, management of diarrhea and use of insecticide treated nets) is computed at an individual level by multiplying the value of the use variable with an individual quality score based on reported components of care received, and thereafter aggregating to obtain a population estimate. The ratio has an associated standard error that is then used to construct the 95% confidence interval.

For the other four interventions described above, quality is estimated from the KSPA as an aggregate for each province with an associated standard error. This is because KSPA was designed and powered to discern estimates at the first subnational level *r* – formerly known as the province. EC is then computed by multiplying Q with the use-need ratio aggregated at *r*. Both these measurements have uncertainties communicated along with the measurements themselves. Thus for each health intervention *j* measured and for each individual *i* observed,

$${EC}_{ij}=\left( \hat{Q}+ \varepsilon_{Q} \right)*\left( \hat{U}+ \varepsilon_{U}| N=1 \right)$$

It is assumed that the two ratios Q and U are independent variables, since they are estimated from independent datasets. In this case, the values of $\varepsilon_{U}$ cannot be observed / measured, but one can calculate the *root mean square* error / the standard error as $\sigma_{U}= \sqrt{\bar{\varepsilon_{U}^{2}}}$ and $\sigma_{Q}= \sqrt{\bar{\varepsilon_{Q}^{2}}}$ . The standard error of the product ($\sigma_{EC}$) can be approximated by the sum of the partial derivatives, applying the propagation of errors law (6) for products of variables.

$\left( \sigma_{ECr} \right)^{2}={\hat{{EC}_{r}}}^{2}* \left( \left( \frac{\sigma_{Q}}{\hat{Q}} \right)^{2}+\left( \frac{\sigma_{U}}{\hat{U}} \right)^{2}+\frac{2\sigma_{QU}}{\hat{Q}\hat{U}} \right)$

Since the variables are independent, their covariance is assumed to be very small ${(\sigma}_{QU} \approx0$). Weighted averages are taken and the standard errors are calculated through Taylor series approximation. The weighted averages formula can be written as below where $w$ represents the sum of the population weights for all regions, and $w_{r}$ is the respective population weight ascribed to the region *r*:

$\hat{EC}_{j}=\sum\left( \frac{w_{r}}{w}*\hat{EC}_{r} \right)$

The partial derivatives with respect to the estimated ECs in each respective region are multiplied by the regional variances obtained, and their sum gives the overall variance.

$\left( \sigma_{ECj} \right)^{2}= \sum\left( \frac{{w_{r}}^{2}}{w^{2}}*{\sigma_{ECr}}^{2} \right)$

In this way the aggregate EC for each intervention is estimated, and presented with the 95% Confidence Interval. Similarly, the aggregate EC for subgroups such as specific socioeconomic groups can be computed by restricting the above calculations to the specified subgroup.

Taylor series approximation is widely used to calculate variance estimates for parametric statistics in large surveys(7). However, care is taken to ensure that the population weights do not overly distort outliers, noting that they are calculated at household level and not individual level. The future direction in this area might involve exploring the performance of other replication-based methods (such as the Jackknife Repeated Replication or Bootstrapping) especially as smaller datasets and datasets with much higher rates of non-response get incorporated for EC estimates.

## References

1. Ng M, Fullman N, Dieleman JL, Flaxman AD, Murray CJL, Lim SS. Effective Coverage: A Metric for Monitoring Universal Health Coverage. PLoS Med. 2014;11:e1001730.

2. Division of Reproductive Health [Kenya]. National Family Planning Guidelines for Service Providers. Nairobi, Kenya; 2010.

3. Lincetto O, Mothebesoane-Anoh S, Gomez P, Munjanja S. Chapter 2: Antenatal Care. In: Lord D, Wake R, Elder L, Grear K, Antayhua A, editors. Opportunities for Africa’s Newborns: Practical data, policy and programmatic support for newborn care in Africa. Cape Town, SA: Mills Litho, Cape Town, South Africa; 2006. p. 51–62.

4. Ministry of Public Health and Sanitation - Govt of Kenya. National Policy on Maternal, Infant and Young Child Nutrition. Nairobi, Kenya; 2012.

5. National Malaria Control Program [Ministry of Health]. Kenya Malaria Strategy 2009 - 2018 (Revised 2014). Nairobi, Kenya; 2014.

6. Ku HH. Notes on the use of propagation of error formulas. J Res Natl Bur Stand Sect C Eng Instrum. 1966 Oct;70C(4):263.

7. Lee ES, Forthofer RN. Analyzing Complex Survey Data. 2nd ed. Thousand Oaks, CA 91320: Sage Publications, Inc.; 2006.
