# Supplementary material for: Determining the effective coverage of maternal and child health services in Kenya, using demographic and health survey data sets: tracking progress towards universal health coverage
Source: Trop Med Int Health. 2017 Feb 7;22(4):442–53. doi: 10.1111/tmi.12841 (PMC5396138; doi:10.1111/tmi.12841)
Supplement: Supplementary file 2 — Appendix S2. Data on specific components used to calculate EC is presented below for each survey period assessed. [file TMI-22-442-s002.docx]

## Supplement 2

## Data on specific components used to calculate EC is presented below for each survey period assessed.

|  |  | 2003 |  |  | 2008-09 |  |  | 2014 |  |  |
| --- | --- | --- | --- | --- | --- | --- | --- | --- | --- | --- |
| MCH Service | Indicator | Use | Need | Quality | Use | Need | Quality | Use | Need | Quality |
| Family planning services | Currently using a modern family planning method | 2772.2 | 5159.3 | 0.593 | 3570.0 | 5760.7 | 0.591 | 10535.9 | 7144.2 | 0.407 |
| Functional antenatal services | Attended 4 or more ANC visits during last pregnancy | 2119.1 | 4051.6 | 0.605 | 1872.3 | 3973.1 | 0.670 | 6865.7 | 3992.9 | 0.446 |
| Skilled delivery and perinatal care | Most recent birth attended to by skilled health provider | 2535.8 | 6102.1 | 0.784 | 2561.5 | 5851.8 | 0.837 | 19563.4 | 12099.4 | 0.513 |
| Breastfeeding during the first 6 months of life | Exclusive breastfeeding | 618.8 | 620.3 | 0.682 | 530.3 | 534.9 | 0.465 | 800.0 | 797.1 | 0.716 |
| Immunisation services | Received complete set of basic vaccines | 707.3 | 1193.5 | 0.580 | 879.9 | 1150.2 | 0.708 | 3865.3 | 3097.2 | 0.556 |
| Management of diarrhoea | Given ORT for management of most recent bout of diarrhoea | 681.7 | 888.5 | 0.381 | 701.7 | 909.1 | 0.503 | 2843.6 | 2326.6 | 0.538 |
| Care seeking for acute respiratory illness and/or fever | Sought medical advice for most recent episode of fever / ARI | 1135.5 | 2496.3 | 0.561 | 710.3 | 1490.4 | 0.692 | 6102.6 | 3571.0 | 0.411 |
| Use of insecticide treated nets | Covered with insecticide-treated net | 1292.2 | 5870.2 | 0.3 | 4181.1 | 5756.8 | 0.740 | 19664.1 | 14815.4 | 0.590 |
