# Supplementary figures and images for: Determining the effective coverage of maternal and child health services in Kenya, using demographic and health survey data sets: tracking progress towards universal health coverage
Source: Trop Med Int Health. 2017 Feb 7;22(4):442–53. doi: 10.1111/tmi.12841 (PMC5396138; doi:10.1111/tmi.12841)

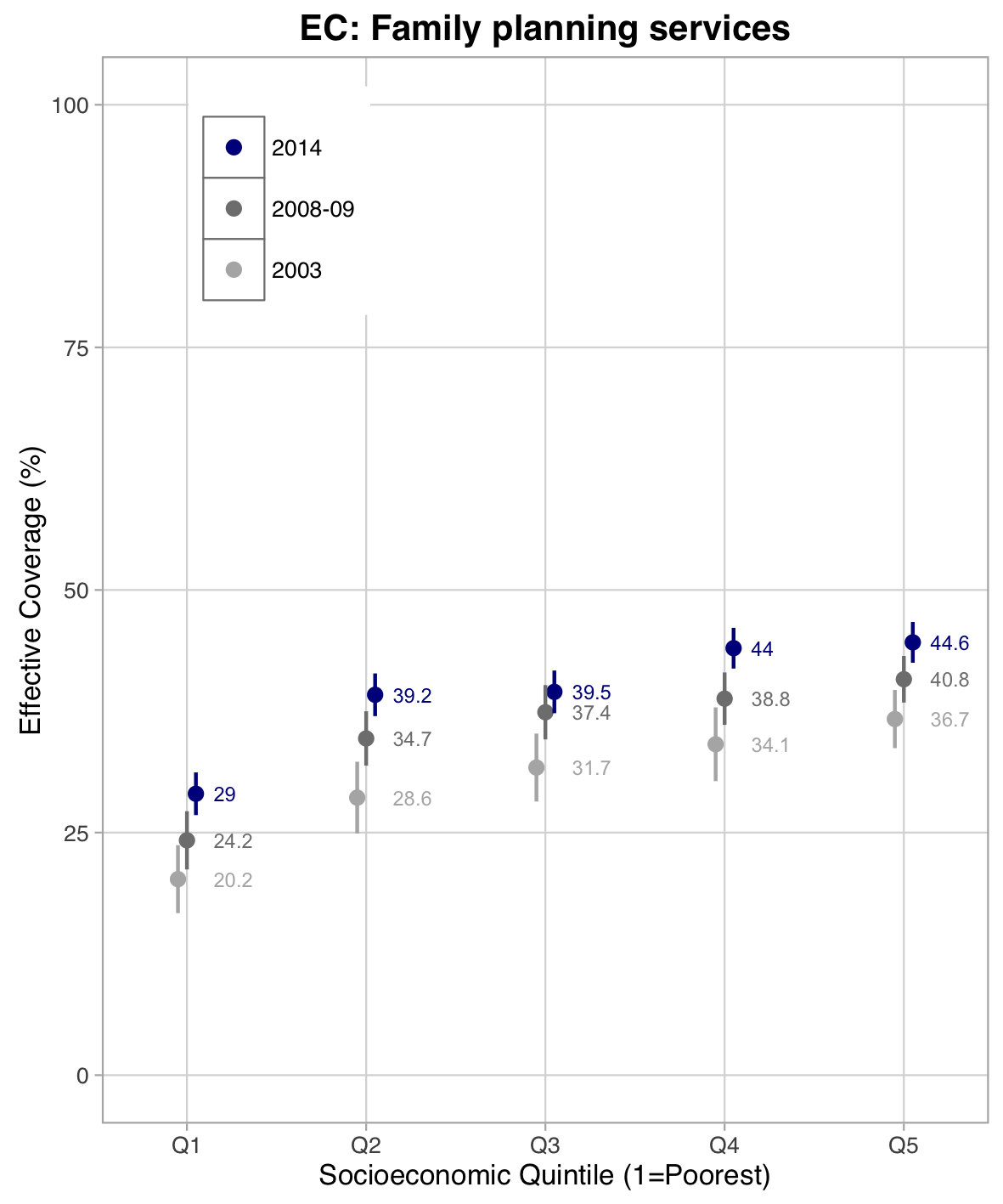

Supplement: Supplementary file 3 — Figure S1a. EC: Family planning services. [file TMI-22-442-s003.tiff]

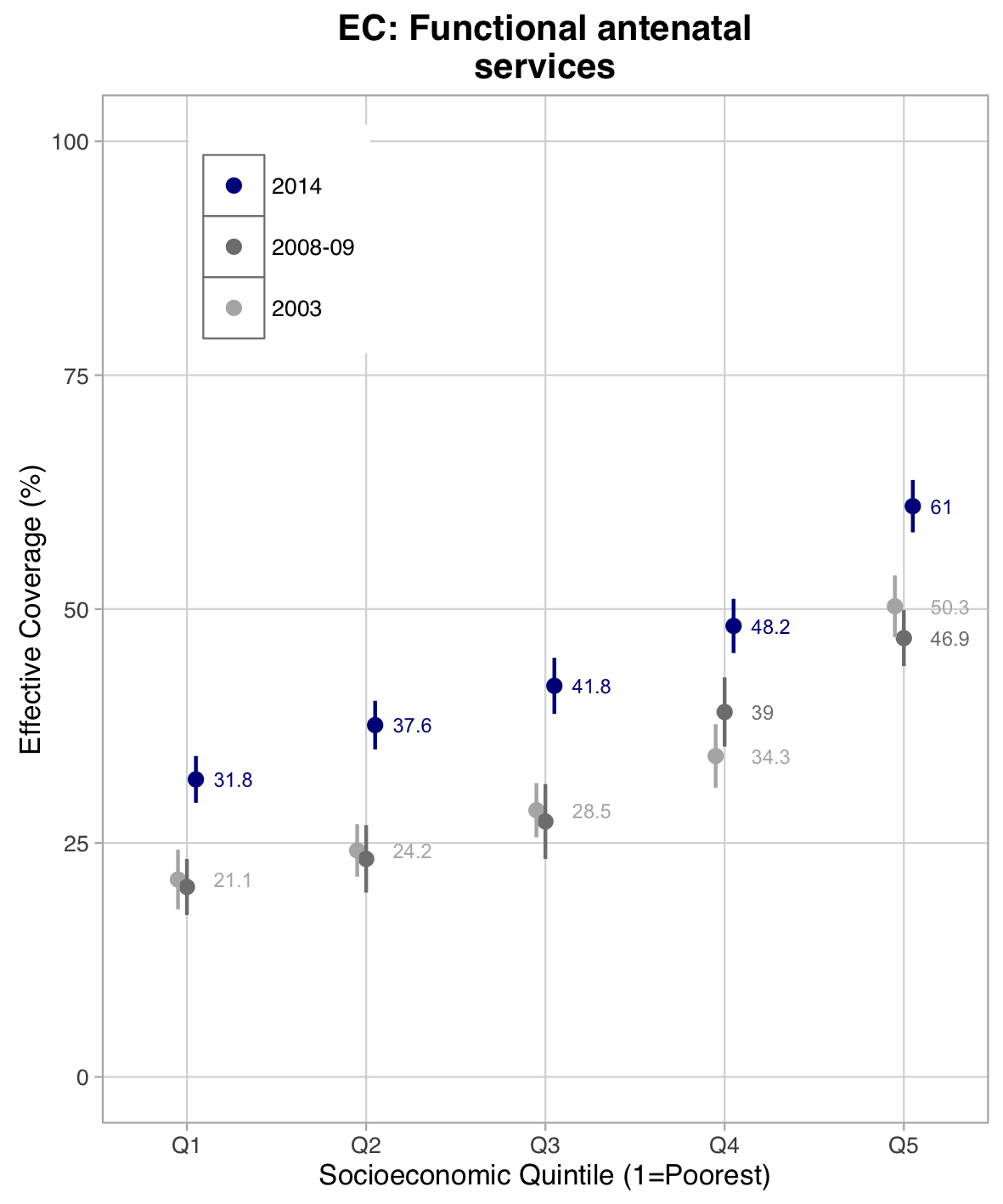

Supplement: Supplementary file 4 — Figure S1b. EC: Functional antenatal services. [file TMI-22-442-s004.tiff]

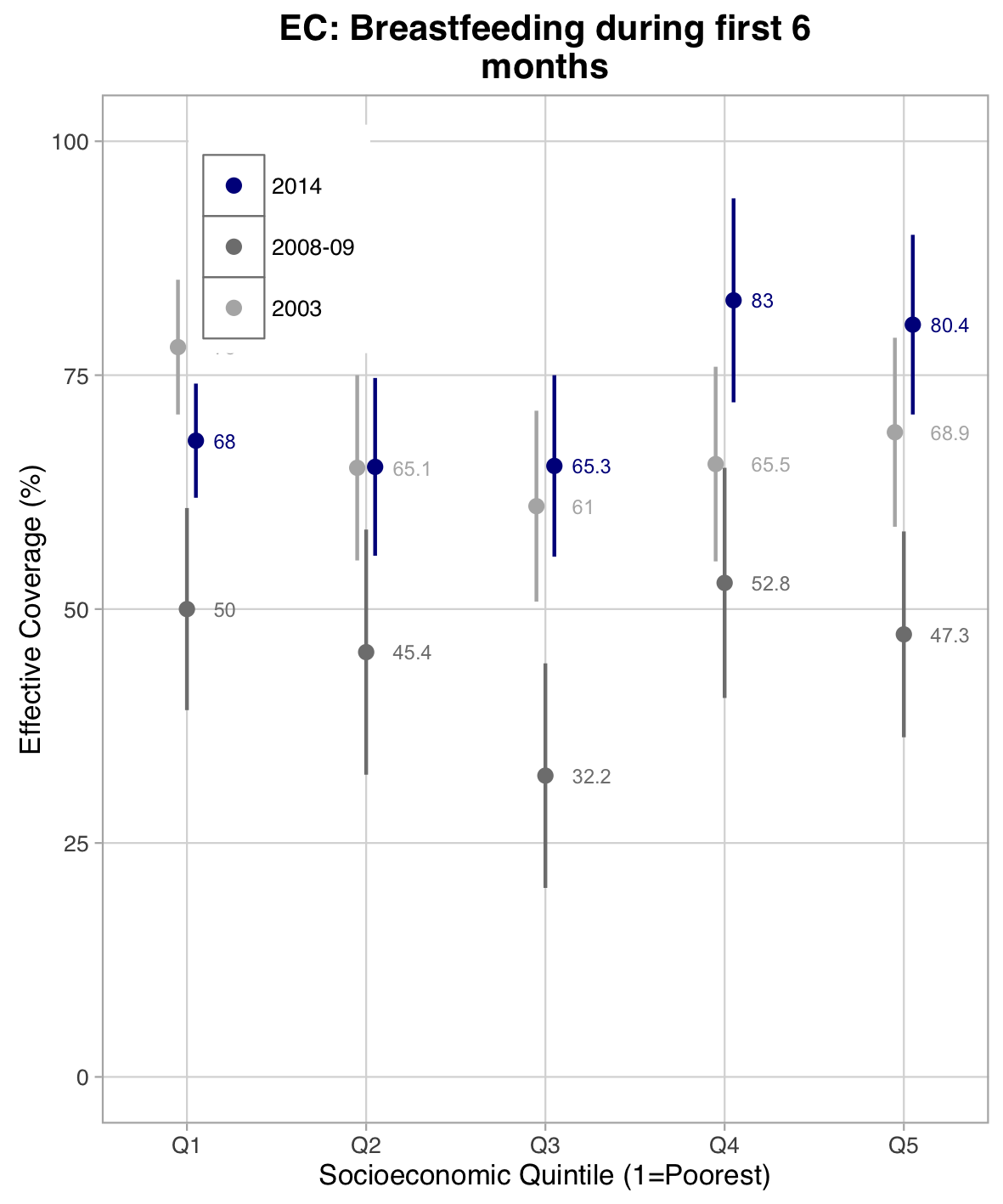

Supplement: Supplementary file 5 — Figure S1c. EC: Breastfeeding during first 6 months. [file TMI-22-442-s005.tiff]

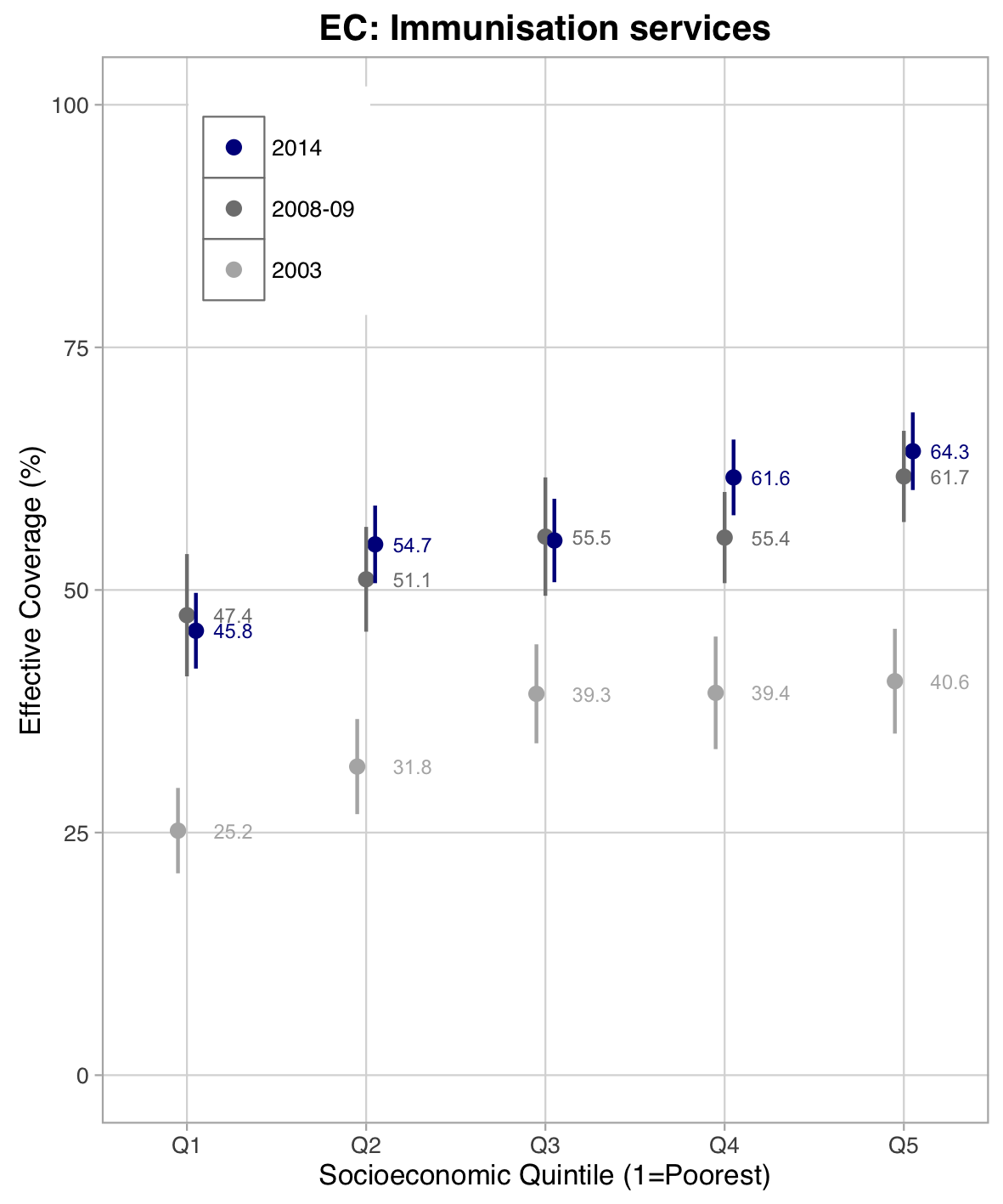

Supplement: Supplementary file 6 — Figure S1d. EC: Immunisation services. [file TMI-22-442-s006.tiff]

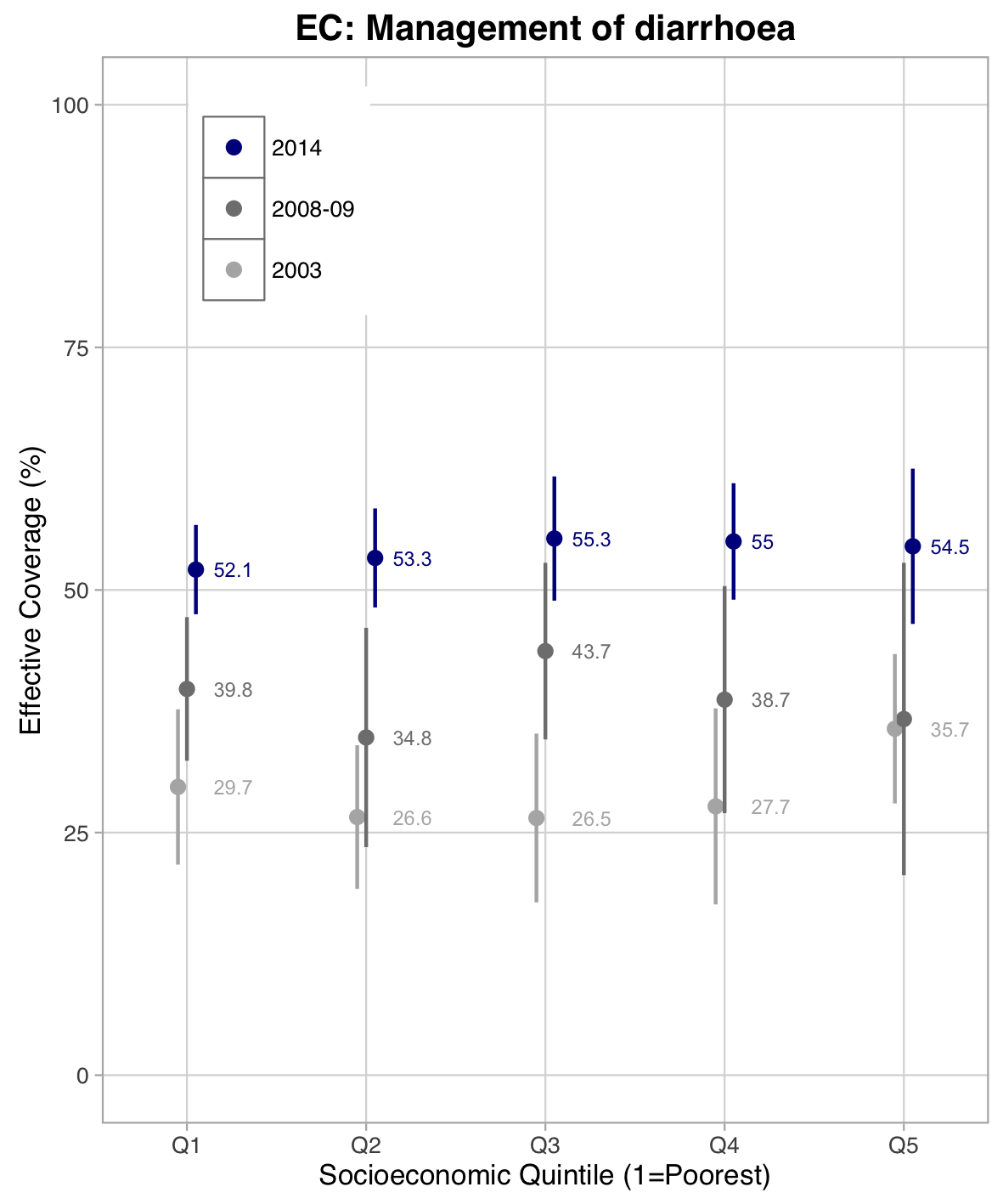

Supplement: Supplementary file 7 — Figure S1e. EC: Management of diarrhoea. [file TMI-22-442-s007.tiff]

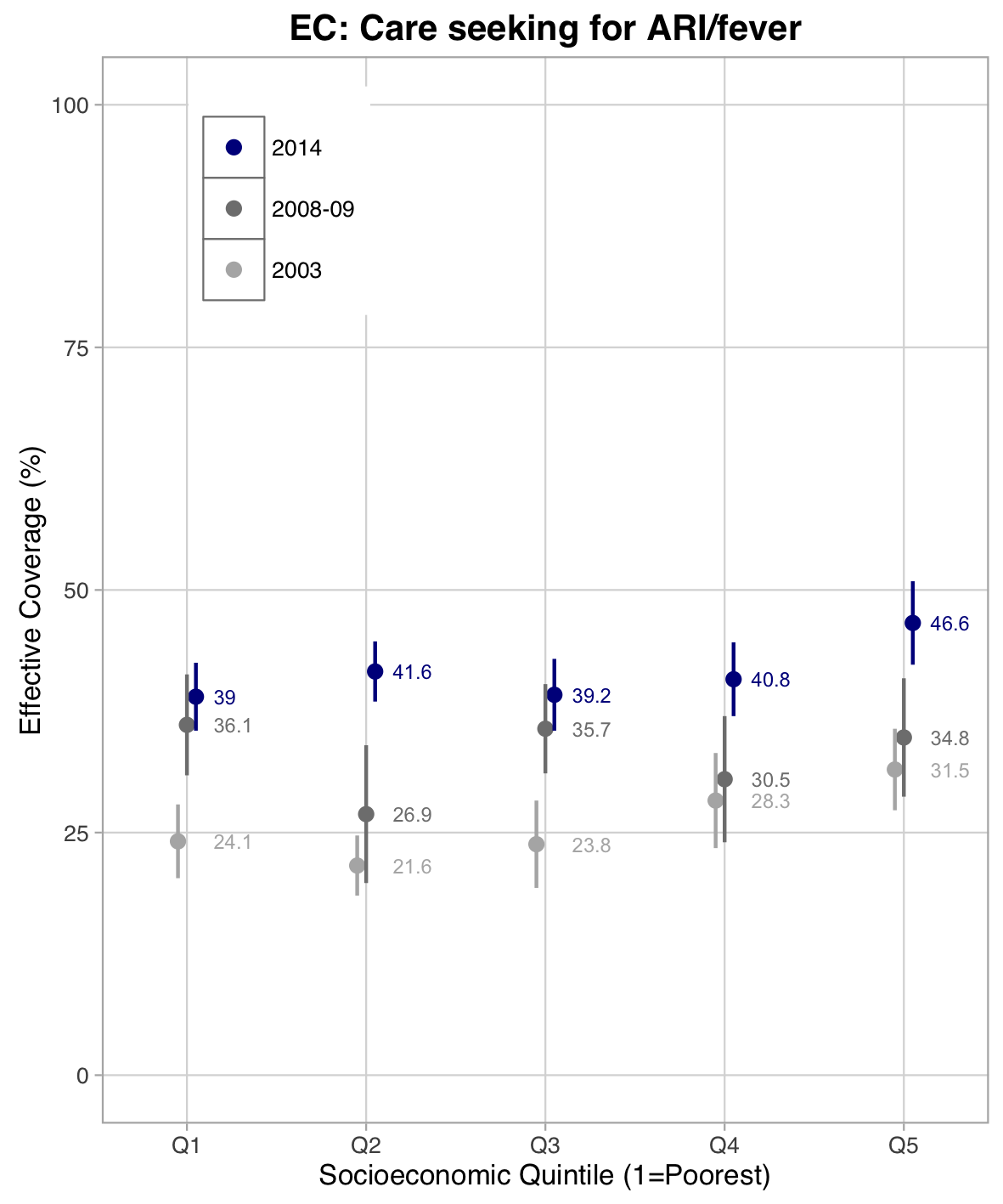

Supplement: Supplementary file 8 — Figure S1f. EC: Care seeking for ARI/fever. [file TMI-22-442-s008.tiff]

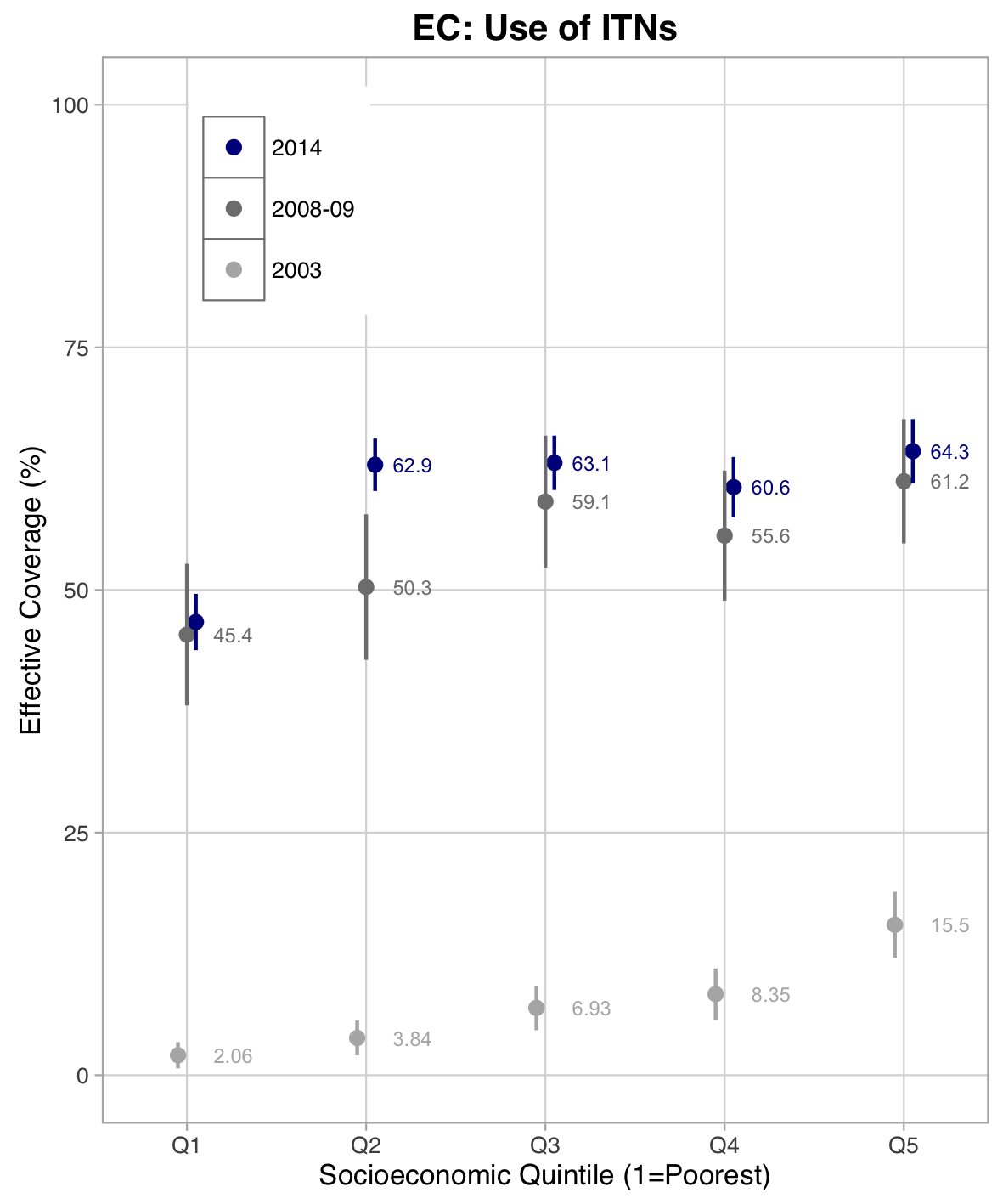

Supplement: Supplementary file 9 — Figure S1g. EC: Use of ITNs. [file TMI-22-442-s009.tiff]

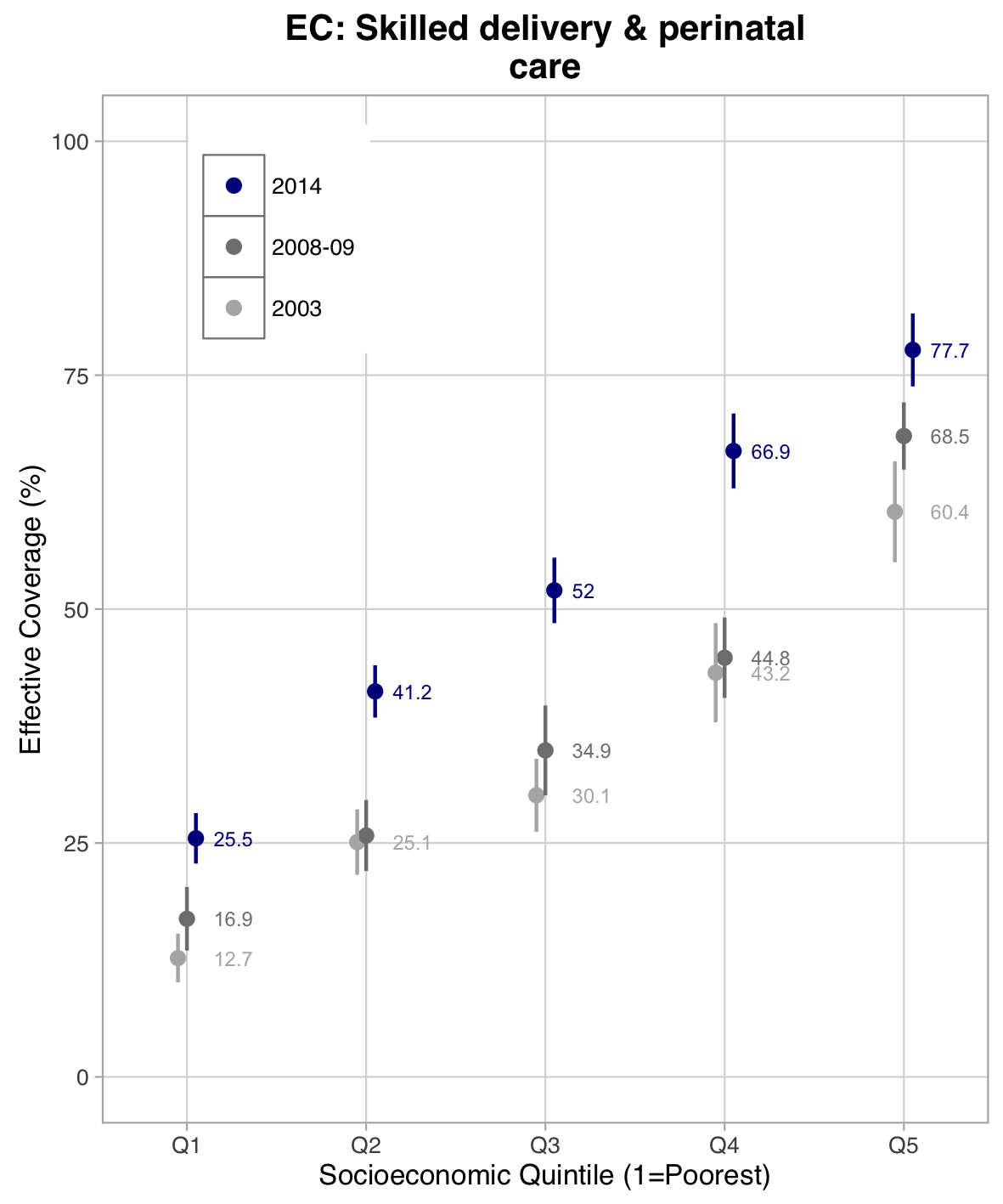

Supplement: Supplementary file 10 — Figure S1h. EC: Skilled delivery & perinatal care. [file TMI-22-442-s010.tiff]
